# Supplementary material for: Serological Biomarkers of Extracellular Matrix Turnover and Neutrophil Activity Are Associated with Long-Term Use of Vedolizumab in Patients with Crohn’s Disease
Source: Int J Mol Sci. 2022 Jul 23;23(15):8137. doi: 10.3390/ijms23158137 (PMC9329899; doi:10.3390/ijms23158137)
Supplement: Supplementary file 1 [file ijms-23-08137-s001.zip › Table S1.pdf]

Table S1. Spearman rank correlation coefficients, determining the association between baseline biomarker levels and disease activity. Data is presented as *ρ* (p-value).

**Biomarker**	**HBI**[a]	**SES-CD**[b]	**Active disease at baseline**
C1M	0.12 (0.545)	0.30 (0.205)	-0.09 (0.638)
C3M	0.08 (0.690)	**0.38 (0.097)**	-0.02 (0.914)
PRO-C3	-0.14 (0.493)	-0.27 (0.255)	**-0.34 (0.081)**
C3M/PRO-C3	0.13 (0.508)	0.40 (0.080)	**0.34 (0.081)**
C4M	0.11 (0.570)	0.30 (0.198)	-0.06 (0.745)
C4G	**0.35 (0.066)**	-0.13 (0.586)	0.12 (0.538)
PRO-C4	0.01 (0.974)	0.21 (0.366)	0.05 (0.800)
C4M/C4G	-0.24 (0.224)	0.21 (0.373)	-0.18 (0.363)
PRO-C4/C4M	-0.12 (0.537)	-0.24 (0.308)	0.26 (0.174)
PRO-C4/C4G	-0.32 (0.103)	0.20 (0.405)	-0.14 (0.491)
C6Ma3	-0.26 (0.185)	0.05 (0.836)	-0.28 (0.151)
CPa9-HNE	-0.12 (0.544)	0.09 (0.701)	-0.05 (0.800)
CPa9-HNE/C4G	-0.30 (0.120)	0.07 (0.777)	-0.11 (0.587)

[a]HBI scores available for 28 patients out of 32 (88%).
[b]SES-CD scores available for 20 patients out of 32 (63%).
