# Supplementary material for: Serological Biomarkers of Extracellular Matrix Turnover and Neutrophil Activity Are Associated with Long-Term Use of Vedolizumab in Patients with Crohn’s Disease
Source: Int J Mol Sci. 2022 Jul 23;23(15):8137. doi: 10.3390/ijms23158137 (PMC9329899; doi:10.3390/ijms23158137)
Supplement: Supplementary file 1 [file ijms-23-08137-s001.zip › Table S2.pdf]

Table S2. Biomarker levels between groups stratified according to whether patients experienced either primary response, secondary response, or no response.

Biomarker	Primary responders (PR) n = 10	Secondary responders (SR) n = 7	Non-responders (NR) n = 15	p-value (PR vs. SR)	p-value (PR vs. NR)	p-value (SR vs. NR)
**Baseline**						
C1M (ng/ml)	**33.7 [26.46–49.21]**	**38.3 [25.38–58.60]**	**108.6 [57.47–148.45]**	0.902	**0.008**	**0.022**
C3M (ng/ml)	10.7 [10.02–14.68]	**8.9 [7.21–11.20]**	**15.2 [13.07–16.44]**	0.189	0.189	**0.006**
PRO-C3 (ng/ml)	7.4 [6.19–8.95]	4.9 [4.06–6.74]	5.7 [5.34. 6.49]	0.308	0.420	0.523
C3M/PRO-C3	1.6 [1.42–1.79]	1.6 [1.45–1.84]	2.6 [1.98. 3.13]	0.774	0.075*	0.075*
C4M (ng/ml)	24.9 [23.42–36.52]	**22.5 [21.08–28.03]**	**36.5 [28.24–46.68]**	0.518	0.109	**0.048**
C4G (ng/ml)	24.1 [15.85–33.60]	19.2 [14.46–21.51]	18.0 [13.56–26.74]	0.688	0.688	0.758
PRO-C4 (ng/ml)	182.2 [175.40–198.77]	182.0 [124.20–212.16]	266.8 [207.20–308.26]	0.663	0.085*	0.068*
C4M/C4G	**0.9 [0.82–1.68]**	1.6 [1.12–1.87]	**2.0 [1.40–2.44]**	0.483	**0.037**	0.483
PRO-C4/C4M	7.4 [6.35–7.98]	6.6 [6.30–7.57]	6.9 [6.51–7.75]	1.000	1.000	1.000
PRO-C4/C4G	7.1 [5.86–11.45]	10.1 [7.22–13.78]	14.5 [9.66–17.12]	0.572	0.099*	0.392
C6Ma3 (ng/ml)	0.7 [0.56–0.81]	0.6 [0.48–0.74]	0.8 [0.68–1.02]	0.602	0.127	0.080*
CPa9-HNE (ng/ml)	**235.5 [202.61–287.24]**	**227.8 [181.74–280.28]**	**401.2 [281.36–452.00]**	0.885	**0.034**	**0.034**
CPa9-HNE/C4G	12.9 [4.73–16.39]	11.6 [9.64–16.28]	18.5 [11.14–29.28]	0.686	0.177	0.424

Significances are post-hoc Holm corrected for multiple comparisons.

*p-values significant before multiple comparison.
