# Supplementary material for: Serological Biomarkers of Extracellular Matrix Turnover and Neutrophil Activity Are Associated with Long-Term Use of Vedolizumab in Patients with Crohn’s Disease
Source: Int J Mol Sci. 2022 Jul 23;23(15):8137. doi: 10.3390/ijms23158137 (PMC9329899; doi:10.3390/ijms23158137)
Supplement: Supplementary file 1 [file ijms-23-08137-s001.zip › Table S3.pdf]

Table S3. Tertile levels for each biomarker. Percentage of long-term users are shown within square brackets for each tertile, along with the corresponding proportions of primary and secondary responders.

Biomarker	Tertile 1 (ng/ml) [%total LTU *(%PR/%SR)*]	Tertile 2 (ng/ml) [%total LTU (%PR/%SR)]	Tertile 3 (ng/ml) [%total LTU (%PR/%SR)]	*P*-value
**C1M**	**11.8 - 38.3 [91 *(55/36)]***	**41.1–107.8 [45 *(27/18)*]**	**108.6–244.4 [20 *(10/10)*]**	**0.004**
**C3M**	**6.9–10.5 [82 *(45/36)*]**	**10.6–15.2 [45 *(18/27)*]**	**15.3–26.5 [30 *(30/0)*]**	**0.049**
PRO-C3	2.9–5.4 [55 *(18/36)*]	5.4–7.2 [36 *(27/9)*]	7.5–16.4 [70 *(50/20)*]	0.302
**C3M/PRO-C3[a]**	**0.7–1.6 [82 *(45/36)*]**	**1.6–2.6 [45 *(27/18)*]**	**2.7 – 5.0 [30 *(20/10)*]**	**0.049**
C4M	16.3–24.5 [82 *(45/36)*]	25.2–36.7 [36 *(18/18)*]	39.2–75.7 [40 *(30/10)*]	0.062
C4G	10.0–15.5 [55 *(27/27)*]	15.8–25.6 [45 *(18/27)*]	25.9–79.3 [60 *(50/10)*]	0.795
**PRO-C4**	**115.6–181.5 [73 *(45/27)*]**	**182.0–260.4 [64 *(36/27)*]**	**266.8 – 522.0 [20 *(10/10)*]**	**0.037**
**C4M/C4G[a]**	**0.5–1.3 [73 *(55/18)*]**	**1.4–2.0 [64 *(27/36)*]**	**2.0–4.8 [20 *(10/10)*]**	**0.037**
PRO-C4/C4M[a]	4.1–6.6 [55% *(27/27)*]	6.7–7.7 [45 *(27/18)*]	7.7–12.9 [60 *(40/20)*]	0.795
PRO-C4/C4G[a]	2.6–7.6 [73 *(55/18)*]	8.4–14.5 [55 *(27/27)*]	14.9–37.0 [30 *(10/20)*]	0.146
C6Ma3	0.4–0.6 [82 *(36/45)*]	0.6–0.8 [36 *(36/0)*]	0.9–1.5 [40 *(20/20)*]	0.062
**CPa9-HNE**	**121.5–231.7 [82 *(45/36)*]**	**235.6–362.3 [55 *(36/18)*]**	**369.5–557.2 [20 *(10/10)*]**	**0.018**
CPa9-HNE/C4G[a]	2.8–10.9 [64 *(36/27)*]	11.6–18.5 [64 *(36/27)*]	18.9–46.1 [30 *(20/10)*]	0.210

Abbreviations: LTU, long-term users; PR, primary responders; SR, secondary responders.
[b]Biomarker ratios with no concentration unit.
